# Genome-Wide Analysis of Antiviral Signature Genes in Porcine Macrophages at Different Activation Statuses

**DOI:** 10.1371/journal.pone.0087613

**Published:** 2014-02-05

**Authors:** Yongming Sang, Wyatt Brichalli, Raymond R. R. Rowland, Frank Blecha

**Affiliations:** 1 Department of Anatomy and Physiology, College of Veterinary Medicine, Kansas State University, Manhattan, Kansas, United States of America; 2 Department of Diagnostic Medicine and Pathobiology, College of Veterinary Medicine, Kansas State University, Manhattan, Kansas, United States of America; Virginia Polytechnic Institute and State University, United States of America

## Abstract

Macrophages (MФs) can be polarized to various activation statuses, including classical (M1), alternative (M2), and antiviral states. To study the antiviral activation status of porcine MФs during porcine reproductive and respiratory syndrome virus (PRRSV) infection, we used RNA Sequencing (RNA-Seq) for transcriptomic analysis of differentially expressed genes (DEGs). Sequencing assessment and quality evaluation showed that our RNA-Seq data met the criteria for genome-wide transcriptomic analysis. Comparisons of any two activation statuses revealed more than 20,000 DEGs that were normalized to filter out 153–5,303 significant DEGs [false discovery rate (FDR) ≤0.001, fold change ≥2] in each comparison. The highest 5,303 significant DEGs were found between lipopolysaccharide- (LPS) and interferon (IFN)γ-stimulated M1 cells, whereas only 153 significant DEGs were detected between interleukin (IL)-10-polarized M2 cells and control mock-activated cells. To identify signature genes for antiviral regulation pertaining to each activation status, we identified a set of DEGs that showed significant up-regulation in only one activation state. In addition, pathway analyses defined the top 20–50 significantly regulated pathways at each activation status, and we further analyzed DEGs pertinent to pathways mediated by AMP kinase (AMPK) and epigenetic mechanisms. For the first time in porcine macrophages, our transcriptomic analyses not only compared family-wide differential expression of most known immune genes at different activation statuses, but also revealed transcription evidence of multiple gene families. These findings show that using RNA-Seq transcriptomic analyses in virus-infected and status-synchronized macrophages effectively profiled signature genes and gene response pathways for antiviral regulation, which may provide a framework for optimizing antiviral immunity and immune homeostasis.

## Introduction

Tissue macrophages (MΦs) comprise a major category of monocytic cells along with blood monocytes (BMs) and dendritic cells (DCs). These cells originate from common monocytic precursors *de novo* or differentiate from BMs [1]. MΦs located in tissues are subsequently polarized into various activation states that are critical for defense responses and regulation of immune homeostasis [1], [2]. The activation status of monocytic cells can be equated to the well-established Th1 and Th2 paradigm in T cells, where MΦs exist as classical M1 and alternative M2 statuses [2], [3]. Four activation statuses of mature MΦs have been well characterized [2]. Classically activated or M1 MΦs develop in response to interferon (IFN)-γ and bacterial products such as lipopolysaccharides (LPS) [2]–[4]; the M2 status was originally ascribed to MΦs alternatively activated by Th2 cytokines interleukin (IL)-4 or IL-13 and is now the M2a subclass. The other subclasses of M2 MΦs include M2b, which is obtained by triggering Fcγ receptors and stimulation of Toll-like receptors (TLRs), and M2c, which is derived from deactivation programs elicited by immunosuppressive cytokines and hormones such as IL-10, glucocorticoids, (GC) and transforming growth factor (TGF)-β [3]–[5].

Macrophages at the different activation statuses undergo immunometabolic changes to differentially express a series of intracellular markers and secretory cytokines/chemokines, which have been linked to regulation of inflammation, tissue repair, T- and B-cell proliferation, phagocytosis and antimicrobial activity, primarily against bacteria and helminthes [4], [5]. However, the interaction between MΦ polarization and viral infection was recently reported [6]–[9]. For example, HIV and respiratory syncytial virus (RSV) have been shown to interact with MФ activation statuses and affect viral pathogenesis and host immune responses [6]–[9]. Stimulation of type I IFN production is pivotal in antiviral responses and leads to the establishment of a cell-autonomous antiviral state (MaV) [10]–[14]. It is well established that subsets of MФs and DCs are major producers of type I IFNs [10], [11]. Understanding the relationships between MФ activation statuses and antiviral states is critical to integrate the antiviral state into the scenario of MФ activation statuses, which have been correlated with immune aspects of inflammation, tissue repair, and overall antimicrobial activity [15]–[19].

Monocytic cells are vital innate immune cells in pigs that provide early immune surveillance and bridge adaptive antiviral immunity [20], [21]; however, few studies have reported the activation status of porcine monocytic cells or how cell activation status relates to antiviral immunity [22], [23]. This omission is significant because many of the most economically important porcine viruses are monocytotropic [17], including porcine reproductive and respiratory syndrome virus (PRRSV). PRRSV is an ideal virus to use to decipher how monocytic cell activation status interacts with antiviral immunity because it directly infects subsets of MΦs and DCs and subverts immune responses in these cells [24]–[26]. Indeed, recent studies postulate that the pathogenesis of PRRS is dominated by the intriguing interplay of PRRSV with monocytic cells [17], [18], [24]–[26]. Similar to other species, porcine monocytic cells, and macrophages in particular, are composed of diverse subgroups of cells with different activation statuses, typically the M1, M2 and antiviral states polarized by various mediators in vitro and in vivo [1]–[5]. These mediators, including pathogen-derived molecules such as LPS and cytokines IFNα/β, IFNγ, IL-4, IL-13, IL-10 and TGF-β, dynamically skew macrophages into diverse activation statuses in response to different pathogenic agents. Classic studies of M1 (induced by IFNγ or LPS) and M2 (induced by IL-4 or IL-13) statuses have been associated with regulation of inflammation, antimicrobial and wound-healing processes; the M2c status induced by IL-10 and TGF-β is anti-inflammatory and was recently associated with the retrieval of immune homeostasis [1]–[5]. In addition, cell antiviral states in response to type I or type III IFNs have been well studied upon viral infection but rarely investigated together with other activation statuses such as in monocytic cells [5]–[14]. We have provided evidence that the antiviral state could be incorporated into the scenario of activation statuses, which together provide a framework for optimizing antiviral immunity and immune homeostasis in monocytic cells [18]. Because most viral infections that target monocytic cells, such as PRRS, are complicated with co-infection or secondary infection by pathogens of other phyla [15]–[19], profiling gene response pathways reacting to viral infection in macrophages at different activation statuses may identify status-specific signature genes for antiviral and immuno-homeostatic regulation. Here we have used a transcriptomic shotgun sequencing (RNA-Seq) procedure in porcine MΦs at different activation statuses to study early gene responses to PRRSV infection [27]–[34]. Our objectives were threefold: (1) genome-wide profile differentially expressed genes (DEGs) in PRRSV-infected MΦs at different activation statuses, including an antiviral state; (2) conduct a pathway analysis of immunometabolic genes in MΦs at different activation statuses altered by PRRSV infection; and (3) identify various gene response pathways for antiviral regulation. Notably, instead of a transcriptomic comparison between infected and non-infected tissues/cells as reported in previous studies [27]–[34], our focus was to examine the comparative transcriptome in macrophages at different activation statuses upon viral infection.

## Materials and Methods

### Animals and Isolation of Primary Cells

All virus and animal procedures were approved by the Kansas State University Biosafety and Institutional Animal Care and Use committees. Conventionally raised, 3- to 5-week-old clinically healthy pigs from a herd without a history of virus infection were used following procedures routinely used in our labs [35]–[38]. Four pigs from one litter were used for collection of primary cells for *in vitro* polarization experiments. The ANOVA Sample Size tool (SigmaPlot11, Systat, San Jose, CA) was used to determine sample size using an expected size (δ) of 0.75, desired power (π) of 0.80 and an error level (α) of 0.05. Blood (20 ml/pig) was collected by jugular venipuncture from anesthetized pigs. Immediately after euthanasia, lungs were lavaged with 300 ml of 10 mM PBS (pH7.4) [36]–[39]. Samples were placed on ice, and peripheral blood mononuclear cells (PBMCs) and MФs were isolated from the heparinized blood and lavage fluid, respectively, within 4 h after collection. Lavage fluids were centrifuged at 400×g for 15 min to collect cells and further isolate MФs by plastic adherence [36]–[39]. Cells were used immediately or cryopreserved in Recovery cell culture freezing medium (Invitrogen, Carlsbad, CA).

### Cell Polarization and Viral Infection

Mediators and conditions for polarization of porcine monocytic cells were applied as described [3]–[5], [13], [17]. In brief, MФs and DCs were stimulated with the mediators of LPS, IFNγ, IL-4, IL-10, IFNα and IFNβ at 20 ng/ml for 30 h (R&D Systems, Minneapolis, MN). All mediators were dissolved in 1×Dulbecco’s phosphate-buffered saline (DPBS, Invitrogen) containing 1% bovine serum albumin (BSA, Fraction V, cold-ethanol precipitated, Sigma-Aldrich, St. Louis, MO) and applied (1∶100) to the cultured cells; only BSA in DPBS was added to cultures of control cells. Cells after polarization were infected with a PRRSV strain (P129-GFP, AF494042) [36] at a multiplicity of infection (MOI) of 0.1 TCID_50_/ml for 5 h and washed twice with fresh culture medium prior to RNA and protein extraction.

### Transcriptomic Shotgun Sequencing

For RNA-Seq, equal quantities of primary alveolar macrophages from three pigs were polarized individually according to procedures described above. Total RNA was extracted from 3×10^7^ cells of each activation status using a column-based RNA/DNA/protein purification kit (Norgen Biotek, Ontario, Canada). RNA integrity and concentration were evaluated with a NanoDrop 8000 spectrometer (NanoDrop, Wilmington, DE) and an Agilent 2100 Bioanalyzer (Agilent Technologies, Santa Clara, CA) to ensure RNA samples with A260/A280>1.8 and RNA integrity number (RIN) >7.0 qualified for construction of sequencing libraries. Messenger RNA purification, fragmentation, construction of sequencing libraries and sequencing were performed using the Illumina Pipeline (BGI Americas, Cambridge, MA). Approximately 25–30 M clean reads per sample were generated for genome-wide transcriptomic analyses. The trimmed reads were further assembled and mapped to the UniGene (http://www.ncbi.nlm.nih.gov/UniGene/UGOrg.cgi?TAXID=9823) and RefSeq (http://www.ncbi.nlm.nih.gov/RefSeq/) collections by performing alignments using BWA software [31], [33]. Using an edgeR procedure, values of reads per kilobase per million mapped reads (RPKM) were generated and used to identify the total number of genes expressed in each porcine sample and DEGs among each comparison [40]. The DEGs between two samples were analyzed based on an algorithm as described [40]. In brief, the P-value corresponds to a differential gene expression test where FDR (False Discovery Rate) was used to determine the threshold of the P-value in multiple tests. The functional classification of genes was carried out through Gene Ontology and KEGG pathway analyses using the DAVID web tool [41], [42]. The dataset was deposited in the NIH Short Read Archive linked to a BioProject with an accession number of SRP033717.

### Confirmation of DEGs using Real-time RT-PCR and a Proteomic Analysis

Real-time RT-PCR assays were used to confirm two families of DEGs revealed by the RNA-Seq protocol, namely the expression of interferon-regulatory factor (IRF) and IL-17 families. Real-time RT-PCR was performed as previously described [35]–[37]. Primers used for RT-PCR assays are listed in Table S1. For confirmation of DEG expression at the protein level, we used two-dimensional difference in gel electrophoresis (2D-DIGE, Applied Biomics, Inc., Hayward, CA). In brief, equal amounts (10 µg) of protein extracts from 2–3 samples of the cells for RNA preparation were labeled with a CyDye dilution (Cy2, Cy3 or Cy5, Amersham, Piscataway, NJ), mixed and simultaneously separated on a single multiplexed 2D gel. After electrophoresis, the gel was scanned with a Typhoon image scanner (GE Healthcare Bio-Sciences, Pittsburgh, PA) to reveal protein spots with increased or decreased intensity compared with the saline control sample. Differences in protein expression were determined with minimum protein volume set at 200 and a 100% presence in all gel images. Only proteins with a twofold or greater difference in protein expression among samples and *p*-values <0.05 (ANOVA) were defined as significant changes and selected. Each spot was verified by manual comparison of three sets of gels before being chosen from a preparative gel and identified by nano LC-MS/MS (Applied Biomics). Scaffold (Proteome Software Inc., Portland, OR) was used to validate MS/MS-based peptide and protein identifications. Protein identifications were accepted if they could be established at greater than 95% probability (assigned by the Protein Prophet algorithm [43]) and contained at least two identified peptides. Genes of 16 randomly chosen proteins that showed a significant difference at the protein level were identified from the RNA-Seq dataset for comparison at the RNA level.

### Antiviral Regulation based on Gene Response Pathways

Gene response pathways significantly altered by PRRSV infection in MФs at different activation statuses were confirmed for their involvement in antiviral regulation using agonists and antagonists to modulate some pathways. Drugs used were inhibitors of the cell epigenetic process, including azacytidine DNA, BIX-01294 and Trichostatin A [44], [45], as well as modulators of AMP-kinase (AMPK) pathways, sodium salicylate (SA) and U18666A [46], [47]. Drugs were diluted in either dimethyl sulfoxide (DMSO; cell culture grade, ATCC, Manassas, VA) or cell culture medium and used to treat cells from 0.01 µM–10 mM after the evaluation of non-significant cytotoxicity effects as described [44]–[47]. Control cells were mock-treated with 0.01% DMSO in culture medium. To evaluate their effect in antiviral regulation, PRRSV infection was conducted simultaneously with drug treatment for 24 h after the cells were washed twice with culture medium [36]. We calculated virostatic effects with the formula (V_t_ –V_i_)/(V_t_–V_0_), where V_t_ represents the value of total/highest occurrence of a viral infection in mock-treated cells, V_i_ is the value obtained from drug-treated cells, and V_0_ is the value from cells without addition of viruses. In addition, viral infectivity was examined after the viral preparation was incubated with the drug solutions for 2 h prior to infecting cells to evaluate the direct effect of drugs on the virus.

#### Data analyses

Relative gene-expression data of real-time RT-PCR were normalized against C_t_ values of the housekeeping gene (GAPDH), and the relative expression index (2^−ΔΔCt^) was determined and compared with the base levels of control samples [36], [37]. Significance analyses pertaining to DEG annotation and pathway analyses were conducted as described using a standard analysis pipeline to determine the *p*-value corresponding to the differential gene expression test and false discovery rate (FDR) and reflect the *p*-value threshold in multiple tests [40]–[43]. Regulation of antiviral activity was evaluated by percentage suppression of viral propagation in cultured cells.

## Results

### Significant DEGs between PRRSV-infected MФs at Different Activation Statuses

Standard analyses were conducted for quality control and to ensure that RNA-Seq data met the criteria for genome-wide transcriptomic analysis [48], [49] (Figure S1 and Table S2). For comparisons of any two activation statuses, we normalized >20,000 DEGs and filtered out 153–5,303 significant DEGs (FDR ≤0.001, fold change ≥2) in each comparison (Figure S1). For example, the highest number of 5,303 significant DEGs was revealed between M1-LPS and M1-IFNγ cells, with 4,257 up-regulated and 1,046 down-regulated, respectively, whereas only 153 significant DEGs were detected between M2-IL10 and mock-stimulated MФs (defined as M0-PBS status) 5 h post-PRRSV infection. Comparing the subtotal DEGs of each status with all others showed that the M1-LPS status had the greatest number (23,843) of significant DEGs, whereas the MaV-IFNα, M1-IFNγ and M2-IL4 statuses had 12,610, 14,965 and 15,005, respectively, and the M2-IL10 and PBS-mock treated cells had the lowest number (about 10,000) of significant DEGs (Figure S1). Therefore, regardless of the overlap of common genes co-regulated at different activation statuses during PRRSV infection, the activation statuses relevant to co-infection with helminthes (M2-IL4) or bacteria (M1-LPS) had many more total DEGs than mock-stimulated MФs upon PRRSV infection (Figure S1).

### DEGs of other Statuses Compared with PBS Mock-stimulated Cells and Potential Signature Genes for Antiviral Regulation of each Activation Status

**Figure 1:**
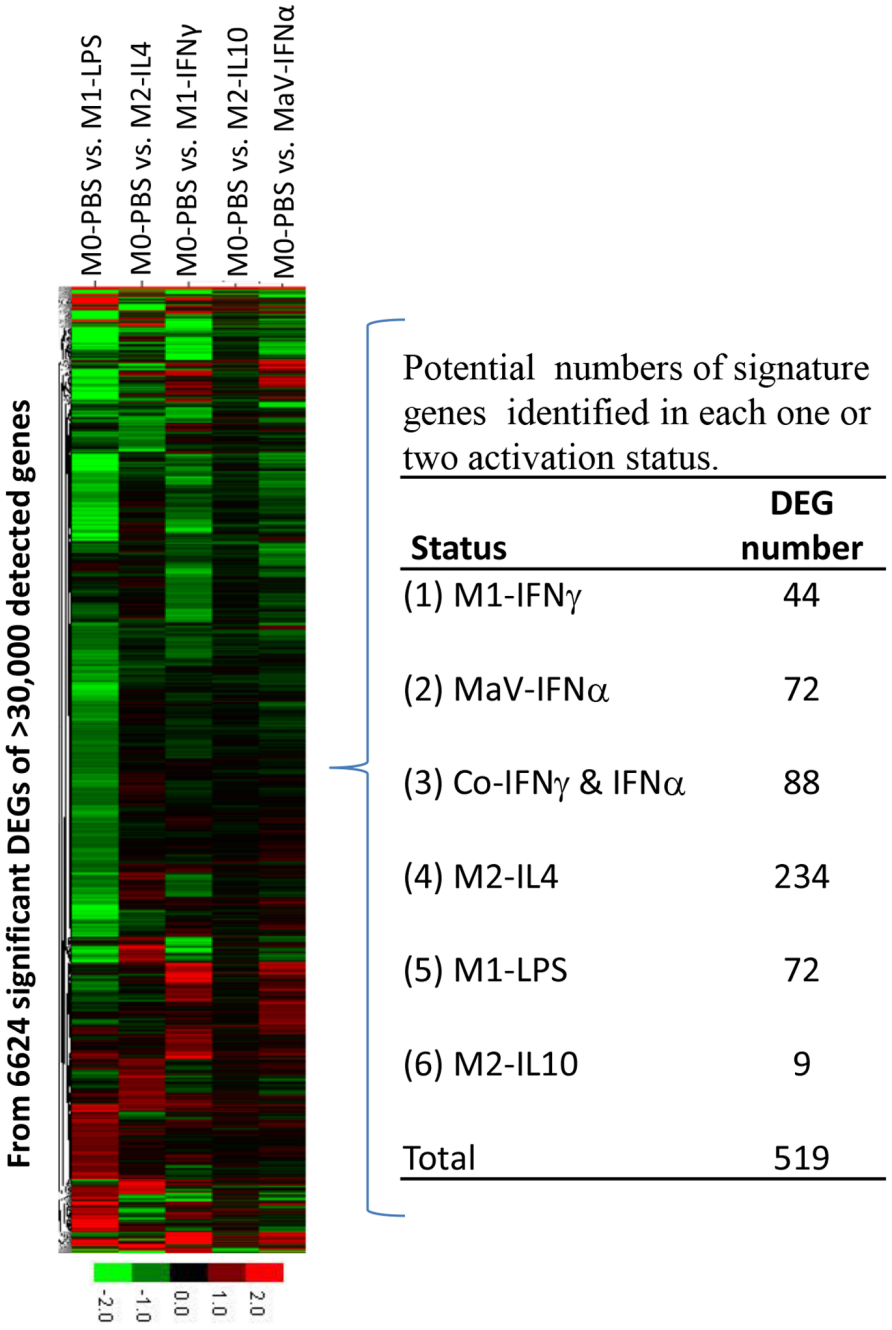
RNA-Seq analysis of DEGs in polarized MФs compared with sham control MФs in PBS post-PRRSV infection. The heatmaps of 6,624 significant DEGs (left) and numbers of potential signature genes were grouped based on significant up-regulation in only one activation status or co-stimulated in two activation statuses (see table at right, and the supplemented results of DEG statistics in Table S2). FDR (false discovery rate) ≤0.001, fold change ≥2 for DEG significant determination. The color scale under the heatmap illustrates the log_2_ (fold change) values shown in the heatmap.

To simplify RNA-Seq data analysis for identification of gene response pathways altered during PRRSV infection in MФs at different activation statuses, we focused our comparisons of DEGs from all five activation statuses on those from the M0-PBS cells. Our combination of all significant DEGs versus the control cells resulted in 6,624 non-redundant genes that were significantly down- or up-regulated in one or several groups of cells at different activation statuses post-PRRSV infection. To profile potential signature genes for antiviral regulation relevant to MФ activation statuses, we clustered DEGs of only those that were significantly up-regulated in each or two activation statuses; for example, we identified 44 and 72 significant DEGs that were up-regulated only in M1-IFNγ and IFNα-antiviral (MaV) states, respectively (Fig. 1). These potential marker genes include some known genes such as CD101, purinergic receptor (P2RY13), vanin 1 (VNN1), and IFN-induced transmembrane protein 1 (IFITM1) as well as unknown transcripts; however, most of these genes have not been studied for their regulation of activation statuses and antiviral immunity (Fig. 1, and data not shown).

### Differential Expression of Transcription Factors (TFs) in Multiple Families and Confirmation of RNA-Seq Data

**Figure 2:**
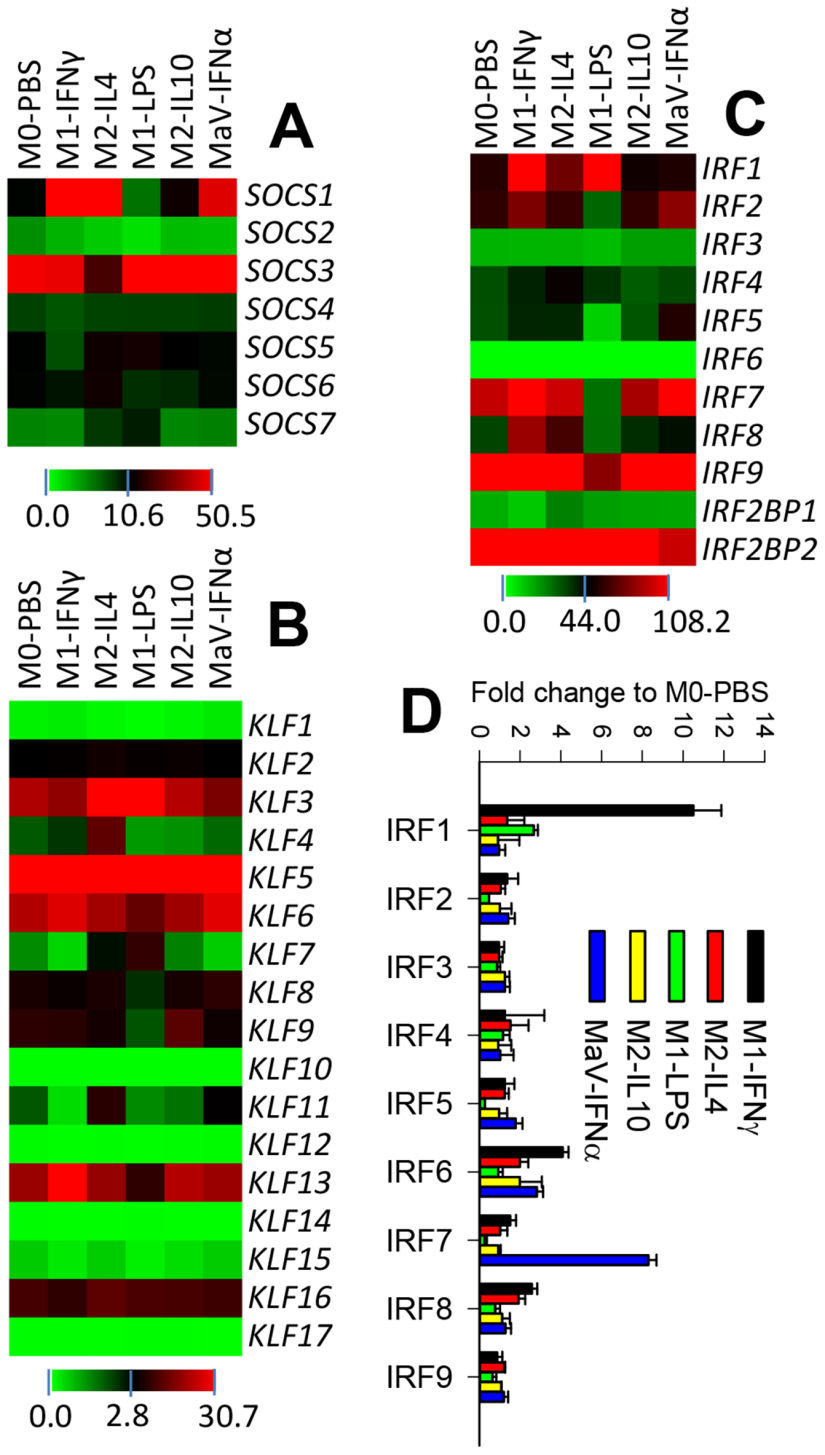
Transcriptomic analysis of selected transcription factor (TF) families. Members of these TF families have been shown to be critical to the regulation of activation status and antiviral activity in murine monocytic cells. Differential expression of TF families of (**A**) suppressors of cytokine signaling (SOCS), (**B**) Kruppel-like factors (KLF), and (**C**) interferon regulatory factor (IRF) in polarized MФs upon PRRSV infection are shown. (**D**) The differential expression of IRF family was verified with a real-time RT-PCR assay (the Y-axis scale indicating fold change to M0-PBS). The color scale under each heatmap illustrates the midpoint and range of reads per kilobase per million (RPKM) values of listed transcripts.

Members of transcription factors in multiple families were identified among DEGs that were significantly regulated at different activation statuses compared with mock-stimulated cells. These transcription factors are in families, such as the suppressor of cytokine signaling (SOCS), Kruppel-like factor (KLF), peroxisome proliferator-activated receptor (PPAR), IFN regulatory factor (IRF) and signal transducer and activator of transcription (STAT), which have been reported to be important in mediation of cell activation and antiviral activity in monocytic cells [50]–[55]. Further curation of the non-filtered DEG datasets (including both significant and non-significant) revealed family-wide coverage of all transcription factors and their differential expression at certain activation statuses post-PRRSV infection. In mice, the developmental bias toward M1-like and M2-like macrophages was associated with the deficiency of *Socs2* and *Socs3*, respectively [50]. Here we showed that the porcine *SOCS2* gene was most down-regulated in the M1-LPS status. In contrast, the *SOCS3* gene was suppressed in the M2-IL4 status 2- to 18-fold compared with expression levels at other statuses (Fig. 2A). In addition, we showed that *SOCS1* was dramatically suppressed in the M1-LPS statuses [51], and *SOCS4* and *SOCS5* were particularly suppressed in the M1-IFNγ status. KLFs, such as KLF2 and KLF4, are another group of transcription factors involved in regulation of inflammatory status of macrophages in humans and mice [52], [53]. We found that porcine *KLF4, KLF7, KLF9* and *KLF13* were particularly up-regulated in the M2-IL4, M1-LPS, M2-IL10 and M1-IFNγ statuses, respectively. In contrast, dramatic suppression of *KLF8, KLF9* and *KLF13* was associated with the M1-LPS status post-PRRSV infection (Fig. 2B). These findings indicate that multiple KLFs may be involved in regulation of the activation status relevant to antimicrobial activity [53].

**Figure 3:**
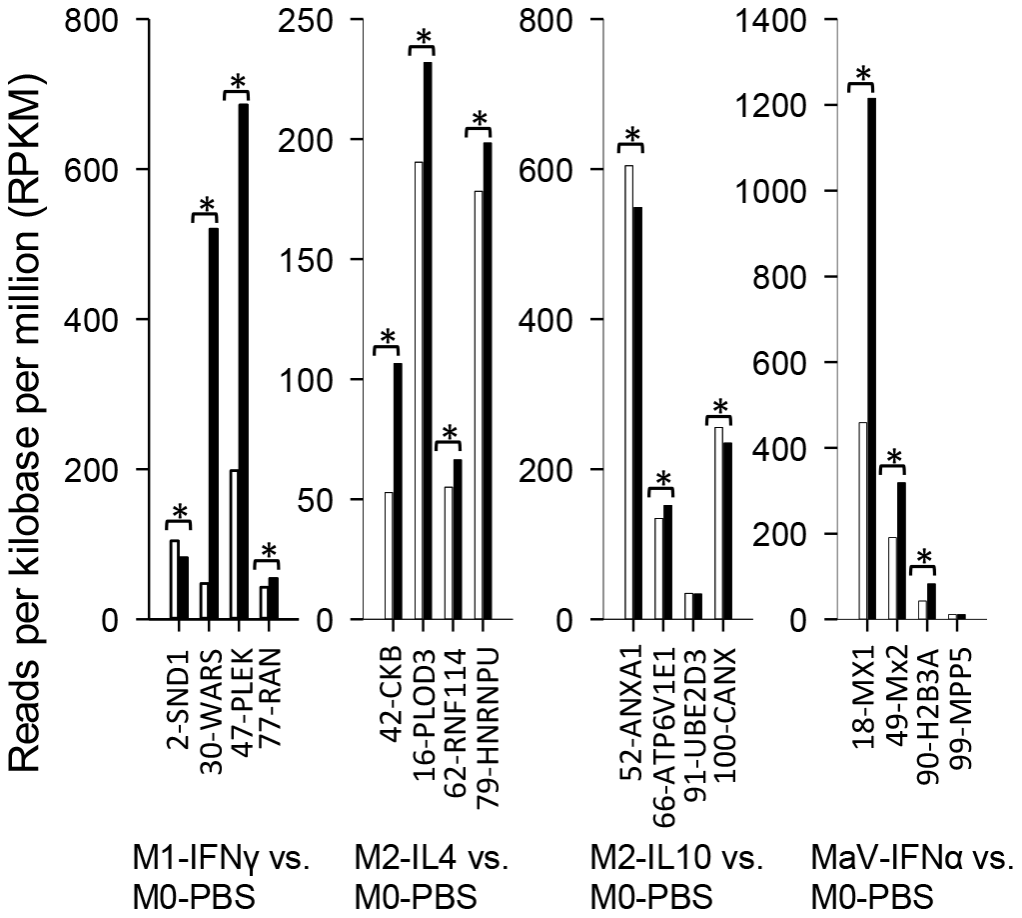
Verification of DEGs at the protein level using a proteomic procedure. Equal amounts of protein from macrophages at different activation statuses were stained with either red or green fluorescent dyes and co-resolved using a 2D-DIGE procedure (Applied Biomics, Inc., Hayward, CA) to isolate protein spots that significantly increased in macrophages at certain activation statuses and to further identify the isolated proteins by nano LC-MS/MS. Of 16 significantly increased protein spots randomly selected across four activation statuses (black bars) compared with the M0-PBS status (the white bars), 12 (75%) protein spots (WARS, PLEK, RAN, CKB, PLOD3, RNF114, HNRNPU, ATP6V1E1, CANX, MX1, MX2, H2B3A) also showed significant up-regulation at the RNA level, with the other four (SND1, ANXA1, UBE2D3 and MPP5) showing a significant increase only at the protein level. *, FDR ≤0.001 of gene expression and protein ratio ≥2. The number before each gene symbol along the X-axis indicates the protein spot mapped in the gel shown in Figure S2. Gene symbol abbreviations: SND1, staphylococcal nuclease domain-containing protein 1; WARS, tryptophanyl-tRNA synthetase; PLEK, pleckstrin; RAN, Ras-related GTP binding C; CKB, creatine kinase B-type; PLOD3, procollagen-lysine, 2-oxoglutarate 5-dioxygenase 3; RNF114, RING finger protein 114; HNRNPU, heterogeneous nuclear ribonucleoprotein U; ANXA1, annexin A1; ATP6V1E1, v-type proton ATPase subunit E 1; UBE2D3, ubiquitin-conjugating enzyme E2 D3; CANX, calnexin; MX, myxovirus resistance gene; H2B3A, histone H2B3A; MPP5, membrane protein palmitoylated 5.

Members of IRFs are implicated in the regulation of a variety of biological processes, including interferon production and modulation of immune cell differentiation [54]. Along with the role of IRF1 and IRF8 in macrophage differentiation, IRF4 and IRF5 have recently been associated with M2-IL4 and M1 statuses, respectively, in murine macrophages [3]–[5]. Our RNA-Seq data showed that porcine *IRF1* was highly stimulated in both M1-IFNγ and M1-LPS statuses, and *IRF8* was stimulated only in M1-IFNγ. Porcine *IRF4* had comparatively constitutive expression but a higher stimulation in the M2-IL4 status than in any other status; similar constitutive expression of *IRF5* was found but dramatic suppression in the M1-LPS status (Fig. 3C). In addition to *IRF5*, porcine *IRF2, IRF3, IRF7, IRF8* and *IRF9* were suppressed more in the LPS-M1 cells than in cells at other activation statuses; however, most were up-regulated in cells at both MaV-IFNα- and M1-IFNγ states. Our RNA-Seq reads also revealed two novel co-repressor molecules for porcine IRF2, *IRF2BP1* and *IRF2BP2* [55]. Despite being relatively constitutively expressed in macrophages of all tested statuses, corresponding suppression of either *IRF2BP1* or *IRF2BP2* was observed with M1-IFNγ and MaV-IFNα statuses, respectively, in which statuses IRF2 had the highest stimulation (Fig. 2C).

To confirm our RNA-Seq expressional analysis, we used real-time RT-PCR assays to re-analyze the expression of porcine IRF genes using RNA aliquots frozen at −80°C. The general expression pattern of all IRF genes, particularly differential expression levels in macrophages at activation statuses different from the PBS-mock control, matched well with the RNA-Seq data (Fig. 2C and 2D). To verify the differential expression of genes at the protein level, we used a 2D-DIGE procedure to isolate protein spots significantly increased in macrophages at certain activation statuses and further identified the isolated proteins by nano LC-MS/MS. Of 16 proteins randomly selected across the four activation statuses, 12 (75%) showed significant up-regulation at both protein and RNA levels, with the other four showing a significant increase only at the protein level (Fig. 3 and Figure S2).

### Gene Response Pathways Significantly Regulated in MФs at Different Activation Statuses upon PRRSV Infection

**Figure 4:**
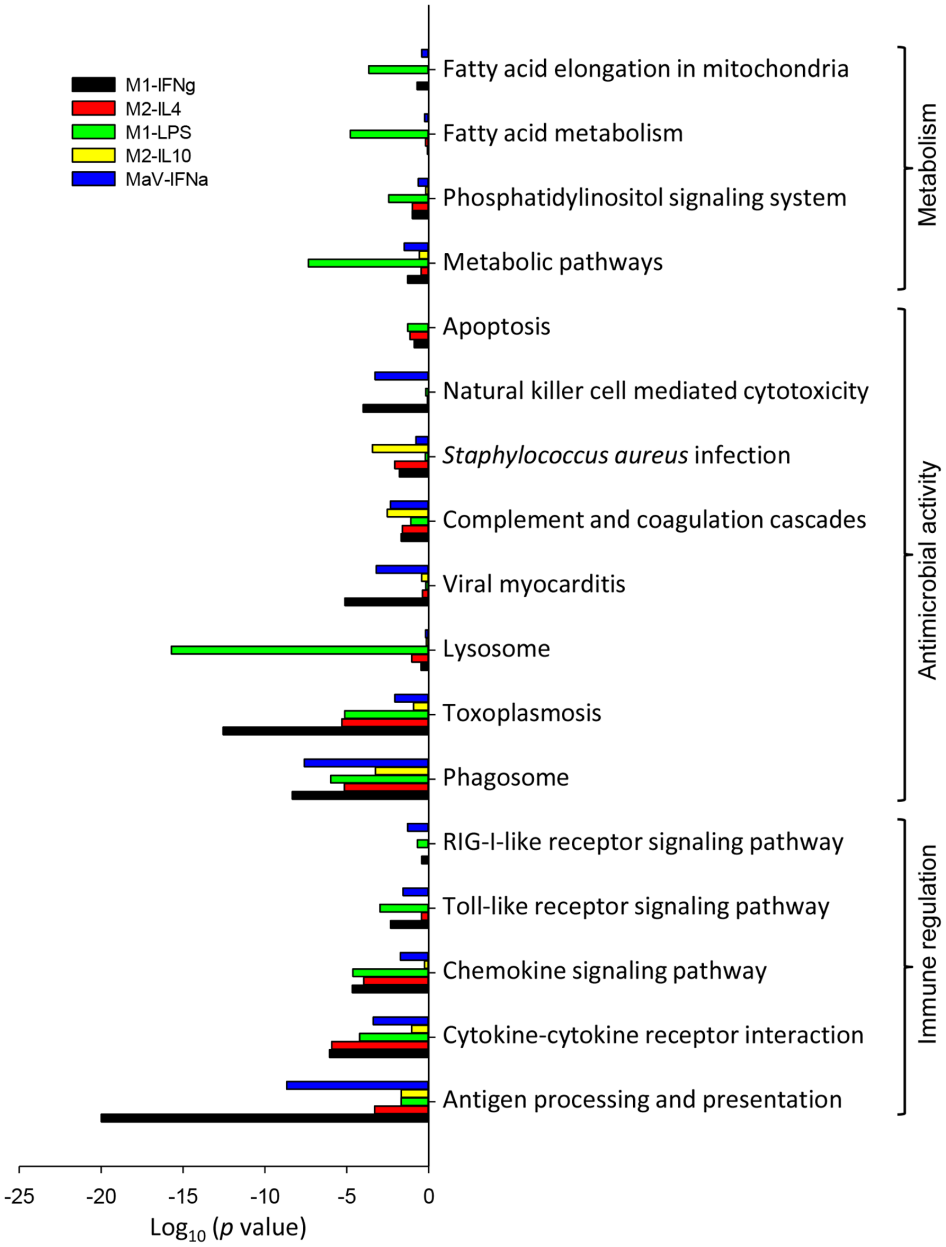
Pathway analysis of DEGs was annotated against the KEGG database. A *p*-value and FDR of <0.05 in the two-sided Fisher’s exact test were considered significant. Selected pathway categories are shown along the vertical axis, and the horizontal axis represents the log_10_ (*p* value) of these pathways showing the significant difference among cells at different activation statuses.

To evaluate the biological and ontological importance of the significant DEGs among different activation statuses, we performed pathway analysis of the DEGs predominately based on the KEGG database (http://www.genome.jp/kegg/). Compared with control cells, significant DEGs in the cells at each activation status were assigned to more than 210 pathways except for the M2-IL10 status, which had the fewest DEGs assigned to 100 pathways. Among these pathways, 20–50 pathways in the cells of each activation status were significantly (*p* and FDR <0.05) enriched by significant DEGs. Most of these pathways belong to immune regulation, antimicrobial response, metabolism and the cytoskeleton system as well as cell development and movement. Figure 4 lists 17 differential pathways that may be important in regulation of macrophage immune function against PRRSV and co-infections. In addition to these pathways, others including chemokine signaling, complement cascade and apoptosis, which exhibited differential responses between PRRSV-infected and non-infected tissues and were previously discovered using gene array-based techniques [27]–[34], we showed that the pathways for antigen processing and presentation, cytokine-cytokine receptor interaction, chemokine signaling and Toll-like receptor (TLR) signaling were among the top immunomodulatory pathways affected by macrophage polarization and that they responded differently to PRRSV infection. The RIG-I-like receptor signaling pathway, which is involved in detection of viral dsRNA during viral replication, was significantly regulated only in cells in the MaV-IFNα state. Correspondingly, the pathways closely relevant to antimicrobial activity, including phagosome, lysosome and antimicrobial reaction against viruses, bacteria and parasites, were significantly regulated. However, pathways related to antiviral response, such as viral myocarditis and natural killer cell–mediated cytotoxicity, were significantly regulated only in cells at two IFN-stimulated statuses, and the regulation of the lysosome pathway, which is critical for bactericidal activity, was prominent only in the M1-LPS status. In contrast, the apoptotic pathway was not significantly regulated and minimally affected in cells at both IL10-regulatory and MaV-IFNα statuses, suggesting potential anti-apoptotic activity during the early phase of PRRSV infection [33]. Metabolic pathways, such as lipid metabolic pathways, were prominently affected in PRRSV-infected macrophages at different activation statuses. LPS-M1 status in particular was more dramatically altered in metabolic pathways, implying synergistic effects of co-infection signaling from PRRSV and bacterial LPS (Fig. 4).

### AMP-activated Protein Kinase (AMPK) Pathway and Epigenetic Regulation are Novel Targets for Anti-PRRSV Regulation

**Figure 5:**
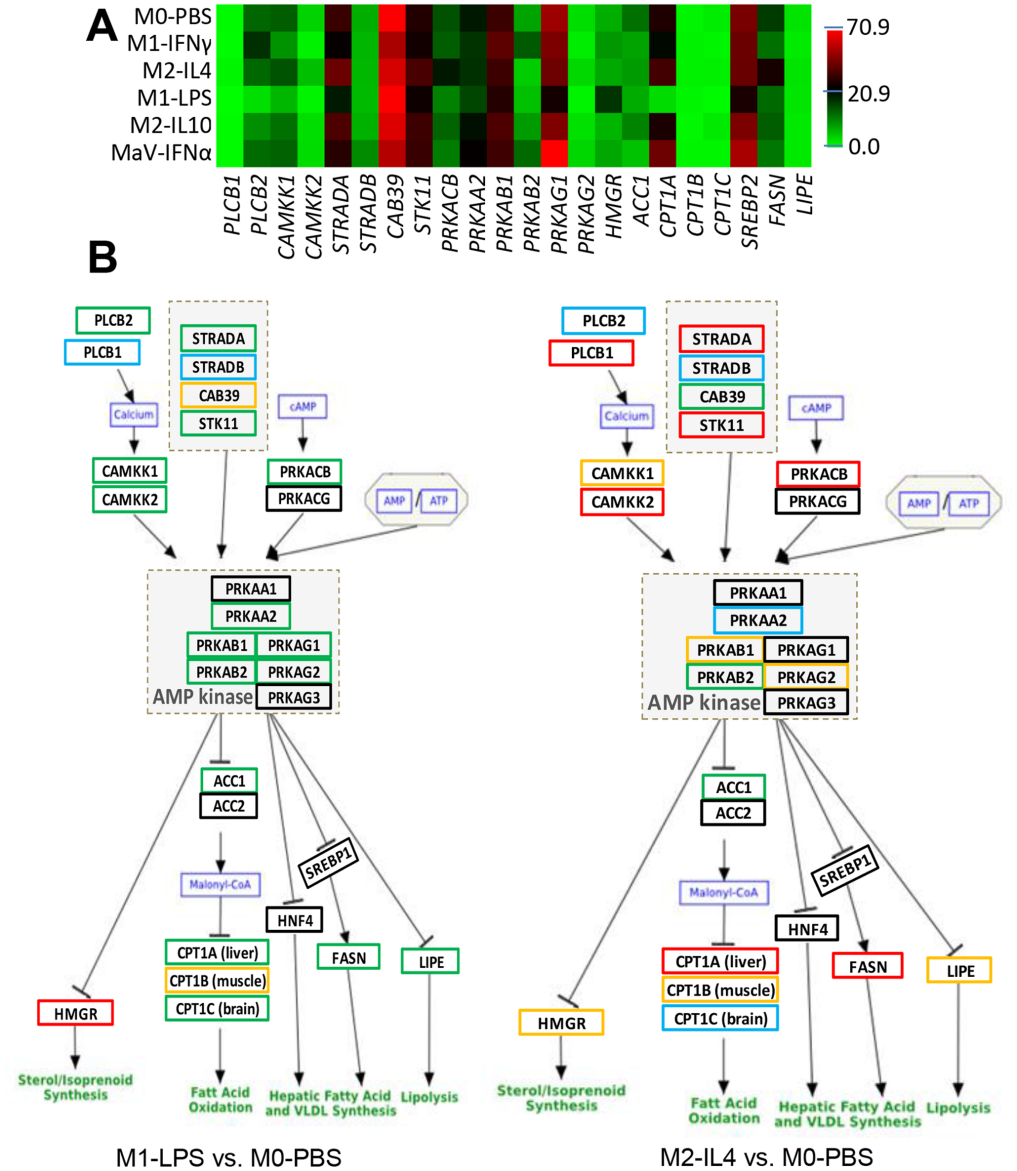
AMPK-mediated pathways for antiviral regulation. (**A**) Heatmap of the subset DEGs in the AMP-kinase pathway, which is critical to control of lipid metabolism, are shown. (**B**) As illustrated in the pathway, most key genes in the M1 statuses (IFNγ- or LPS-induced) or IFNα-antiviral state (MaV) were differentially regulated, leading to a general suppression of lipid metabolism in contrast to a general increase in the M2-IL4 status. In addition, the illustration of AMPK-mediated pathways in other statuses is presented in Figure S3 together with the dose-dependent suppression of PRRSV infection by two AMPK-pathway activators. *Legend:* green-line box, significant suppression; blue-line box, non-significant suppression; red-line box, significant up-regulation; yellow-line box, non-significant up-regulation; and black-line box, non-significant detection. *p* (FDR) <0.001, fold change ≥2 for significant determination.

AMPK consists of a catalytic α subunit and regulatory β and γ subunits, and plays a key role as a master regulator of cellular energy homeostasis through sensing the intracellular AMP:ATP ratio [46], [47]. AMPK activation positively regulates signaling pathways that replenish cellular ATP supplies, including fatty acid oxidation and autophagy, and negatively regulates ATP-consuming biosynthetic processes including gluconeogenesis, lipid and protein synthesis. Because of the discovery of significant modulation of multiple metabolic pathways, particularly lipid/fatty acid metabolism downstream of AMPK-signaling, we sought to annotate the AMPK pathway in detail. As shown in Fig. 5, of 22 genes at the center of AMPK signaling, most were within the list of significant DEGs (Fig. 5A). Dramatic differences were detected between M1 and M2 statuses. As illustrated in Fig. 5B, 13 of the AMPK and AMPK-regulated genes were significantly down-regulated in cells at the LPS-M1 status; in contrast, most were significantly up-regulated in the IL4-M2 status. Consequently, we observed differential regulation of multiple lipid metabolic pathways during pathway analysis (Fig. 4). Because AMPK signaling is critical for immunometabolic regulation and has not been implicated in antiviral response against PRRSV infection, we further validated the involvement of the AMPK-mediated pathway in anti-PRRSV response using agonists of AMPK-signaling. As shown in Figure S3, both AMPK activators, salicylate (SA) and U18666A, significantly suppressed PRRSV infection in MARC-145 and porcine monocyte-derived dendritic cells (mDCs) at the tested doses without causing detectable cytotoxicity [46].

**Figure 6:**
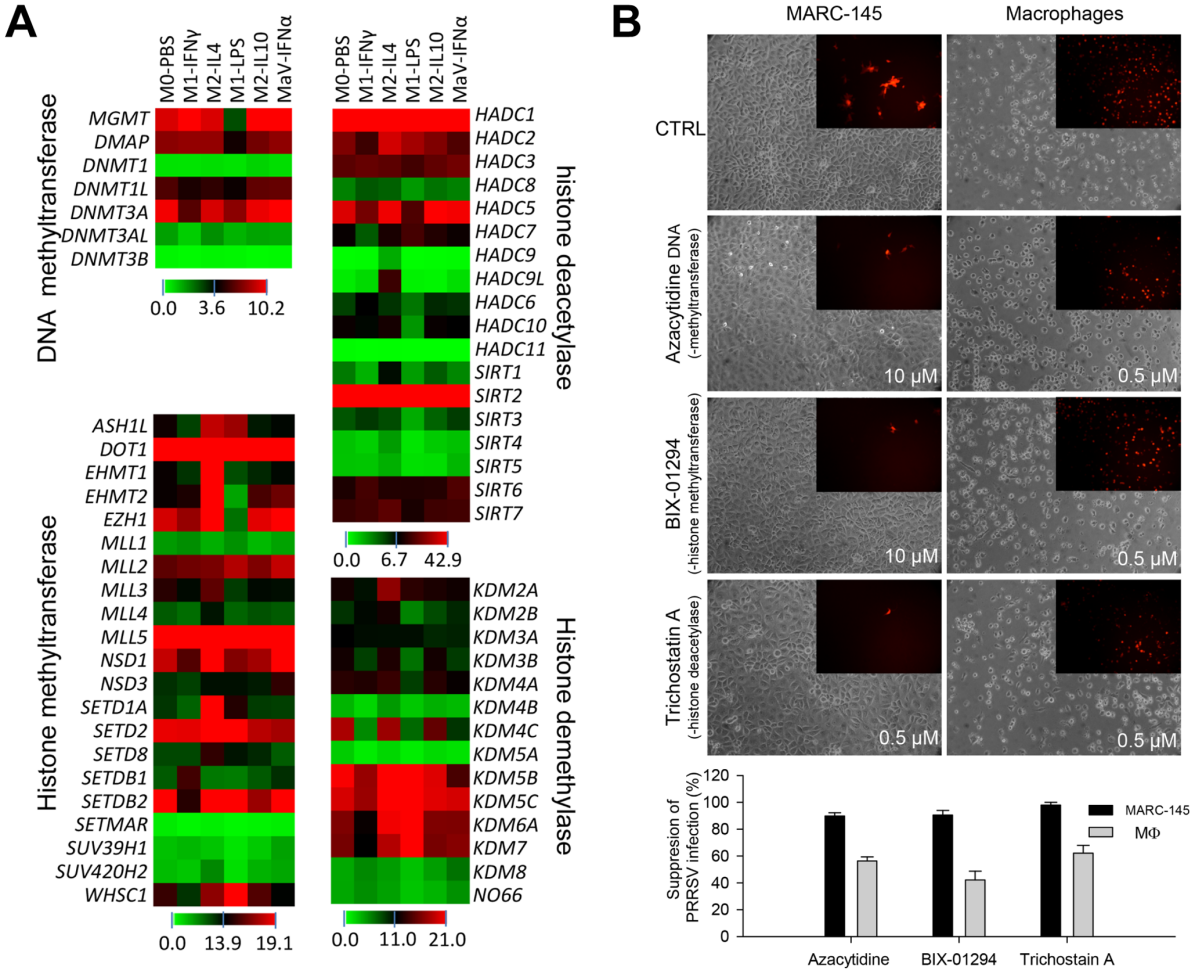
Epigenetic mechanisms for antiviral regulation. (**A**) RNA-Seq analysis of DEGs encoding key enzymes in epigenetic regulation. The heatmaps display family-wide collections of genes encoding DNA/histone methyltransferases, histone deacetylases and histone demethylases. In addition to differential expression analysis, these data also revealed family-wide transcription evidence of most of these porcine epigenetic genes at the mRNA level for the first time. (**B**) Suppression of PRRSV infection by epigenetic inhibitors at optimized concentrations in MARC-145 cells and porcine MФs. The fluorescent micrographs (inset) show infected cells using a DsRed-labeled PRRSV, whereas the larger bright-field images show cell phenotypes of non-visible cytotoxic effects. The micrographs represent one of three replicates. Summary data are presented below the images.

In addition to the AMPK-signaling pathway, annotation of DEGs in our RNA-Seq data revealed family-wide differential expression of genes involved in epigenetic regulation, which is defined as non-genetic alterations critical to biological adaptability to environmental stimuli. Epigenetic traits are tightly regulated by two major epigenetic modifications: chemical modifications to the cytosine residues of DNA (DNA methylation) and histone proteins associated with DNA (histone modifications) [44], [45]. Studies of epigenetic regulation to potentiate antiviral responses have recently emerged [56]. We showed that multiple genes encoding enzymes responsible for catalyzing epigenetic regulation, including DNA methyltransferases, histone methytransferases, histone demethylases and histone deacetylases, were significantly differentially expressed in PRRSV-infected MФs polarized at different activation statuses. Expression of multiple genes important to epigenetic regulation increased significantly at the M2-IL4 status and decreased at the M1-LPS status, which included DNA methylatransferase genes *DNMT3A* and *DNMT3AL*, the histone methyltransferase-related genes *ASH1*, *EHMT1, EHMT2*, *EZH1, MLL3, MLL4, SETD1A, SETD8* and *SUV420H2*, as well as *HADC2, HADC9(9L), SIRT1* and *KDM2A* genes responsible for other histone modification processes (Fig. 6A). Because we previously showed that the M2-IL4 status of porcine macrophages was associated with a moderate increase in PRRSV infection, we investigated whether epigenetic mechanisms could be exploited for anti-PRRSV regulation in cells permissive to PRRSV infection. As shown in Fig. 6B, using dosages that did not cause detectable cytotoxic effects (data not shown), inhibitors of DNA methytransferases (azacytidine DNA), histone methytransferases (BIX-01294) and particularly histone deacetylases (Trichostatin A), were all effective in suppressing PRRSV infection in both porcine macrophages and, in particular, MARC-145 cells. Although 40–60% suppression in PRRSV infection was found in porcine primary macrophages, more than 90% suppression of PRRSV infection was observed using various epigenetic inhibitors in the MARC-145 cells (Fig. 6B).

## Discussion

Porcine reproductive and respiratory syndrome (PRRS) remains one of the most globally devastating swine diseases and a challenge to both porcine immunology and vaccinology [17], [24]–[26]. The viral etiology of PRRS, PRRSV, is an enveloped RNA virus with a high mutation rate and capability of evading porcine immune responses [17], [18], [24]–[26]. The primary infection routes of PRRSV are respiratory and reproductive tracts, where monocytic lineage cells, particularly subsets of MФs (alveolar and intravascular) and DCs (inflammatory monocyte-derived mDCs), are highly permissive to the virus [24]–[26]. Direct infection plus other mechanisms enable PRRSV to subvert critical immune responses exerted by monocytic cells, which include suppressing cell antiviral signaling, diverting cytokine production and action, directing cytolysis, suppressing phagocytic and microbicidal activity, as well as reducing antigen presentation to T cells [17], [18], [24]–[26]. This innate immune aberration further leads to inefficiency in bridging adaptive immunity, which jointly causes immunosuppression of primary PRRSV infection as well as co-infections or secondary infections by other pathogens [17]. Clearly, monocytic cells play a dominant role in PRRSV pathogenesis. Thus, profiling signature genes and gene response pathways in functional subsets of porcine monocytic cells is critical for understanding leukocyte biology and searching gene-targeted measures to control monocytotropic infections [17] by pandemic pathogens including PRRSV.

Using a laboratory-attenuated PRRSV strain [35], we investigated the early gene response (5 h post-infection) in porcine macrophages at activation statuses polarized *in vitro*. During interaction with the virus in this early phase, macrophages maintained their original polarization and cell integrity without any observed loss of viability, which allowed us to investigate the early immunometabolic responses prior to cellular exhaustion by viral replication and release. RNA-Seq instead of a microarray procedure was chosen based on the maturity of this next-generation sequencing procedure and the potential to detect unidentified transcripts [48], [49]. Although previous studies have used gene-array or RNA-Seq techniques to perform genome-wide transcriptomic analyses in PRRSV-infected tissues or primary cell collections [27]–[34], our study is the first to investigate antiviral responses in synchronized macrophage subsets. In addition, pathway analysis using profiled DEGs in synchronized macrophages revealed discoveries that possibly were masked in multiple cell types in tissues and would be necessary for *in vivo* antiviral regulation targeting monocytic cells. Furthermore, we verified that some gene response pathways could be exploited as potential targets for antiviral regulation, which, to our knowledge, has not been validated by previous reports relevant to genome-wide gene mapping of anti-PRRSV responses [27]–[34].

RNA-Seq analyses have been reported to require more than 18M reads for each sample to reach a saturated state for novel gene discovery and expressional analysis [48], [49]. To meet this criterion for genome-wide transcriptomic analysis, we obtained approximately 30M reads for each sample with >99% clean reads; the majority could be mapped to current gene or genome databases. Indeed, our quality control assays, including saturation analysis as well as distribution and coverage analyses in reference genes and genomic scaffolds, displayed an RNA-Seq dataset well qualified for cross-sample transcriptomic analysis (Figure S1). Bioinformatic analyses of DEGs revealed thousands of significantly differentially expressed genes in cells at different activation statuses. As expected, viral infection in cells at M1-LPS and M2-IL4 statuses, which mimicked the co-infection stimuli of a virus with bacteria or helminthes, respectively, had the greatest number of DEGs (Fig. 2 and Figure S1). The significantly lower and comparable DEG numbers in both M2-IL10 and PBS non-stimulated statuses are in agreement with the immune homeostatic regulation role of the M2-IL10 status and the relatively naïve status of our primary cells without stimulation (Fig. 2 and Figure S1) [2]–[5].

Compared with the M0-PBS cells from the mock stimulation, we identified 6,624 DEGs that were significantly up- or down-regulated in cells at five activation statuses. To profile signature genes with potential as markers for phenotyping macrophages and antiviral regulation, the status-specific up-regulated genes related to each activation status post-PRRSV infection were clustered. Ten to several hundred significant DEGs that were up-regulated at only one or two statuses were pooled. Preliminary identification of these potential signature genes identified some known genes and unknown transcripts up-regulated in M1-IFNγ and MaV-IFNα statuses. Most of these genes could be candidates but remained elusive in regulation of antiviral immunity and activation statuses. Only nine potential signature genes were identified for the M2-IL10 cells, which were at a regulatory status and may be critical to restore immune homeostasis after infection (Fig. 2) [2]–[5].

Transcription factors, including members of IRF, SOCS and KLF families, have been implicated in the regulation of development and activation process of monocytic cells in humans and mice [53]–[57], but they have been seldom studied in pigs. Family-wide coverage of multiple families of transcription factors were scrutinized using our RNA-Seq reads. This approach not only provided transcriptomic evidence of these porcine genes, but also allowed cross-species immunogenetic comparison. For example, murine orthologs of SOCS2, SOCS3, KLF2, KLF4, IRF4 and IRF5 have been implicated in regulation of monocytic cell activation [51]–[55]. Our DEG analyses among cells at different activation statuses showed that porcine orthologs of these transcription factors may conserve their role in regulation of macrophage activation. Our transcriptomic data also revealed that some other members of these transcription factors could play significant roles in macrophage development and activation, which include *SOCS1, KLF7, KLF9, KLF13* and *IRF2* being specifically down-regulated at the LPS-M1 status as well as *KLF9* significantly up-regulated only at the IL10-regulatory status. The family-wide profile of multiple gene families indicated that the quality of our RNA-Seq data fulfilled the criteria for genome-wide transcriptomic analysis. Following suggested procedures, we verified IRF expression by RT-PCR assay and matching the general expression patterns over IRF expression in porcine macrophages at different activation statuses. Furthermore, we verified the expression of 16 randomly chosen genes at the protein level, showing that most were in agreement at both protein and RNA levels (Fig. 2D and Fig. 3). Four genes showed a significant increase at only the protein level, suggesting that some gene stimulation was regulated through enhancing translation efficiency rather than increasing new transcripts (Fig. 3 and Supplement Fig. S2).

Pathway analyses of significant DEGs based on the KEGG database clustered them into 5–30 pathways significantly regulated at each activation status compared with the control. The M2-IL10 status had only five pathways that were significantly regulated compared with more than 20 in cells at other activation statuses during the early phase of PRRSV infection. These five significant pathways of the M2-IL10 status regulate toward homeostatic retrieval and may represent the essential responses of porcine macrophages to PRRSV infection. Antigen processing and presentation was the most significant pathway regulated in PRRSV-infected macrophages at both IFNγ-M1 and IFNα-MaV states, indicating that macrophages at these two states were adapted for stimulation of both innate and adaptive immunity [10]–[13]. Macrophages at the LPS-M1 status had most the pathways involved in immune and antimicrobial regulation, and metabolic pathways, in particular, were significantly regulated the most in this status post-PRRSV infection. This finding implies that more profound responses were regulated by stimuli from bacterial and viral co-infection. Notably, our RNA-Seq data using PRRSV-infection of synchronized macrophages revealed many more pathways potentially associated with PRRSV infection, which includes significant pathways elucidated previously by microarray procedures, including antigen processing and presentation, Toll-like receptor signaling, complement and coagulation cascades and chemokine signaling pathways (Fig. 4) [27]–[34].

Two other pathways, which we annotated in detail and verified their involvement in antiviral responses against PRRSV infection, were the AMPK-mediated pathway and the epigenetic regulation pathway [44]–[47]. The multi-subunit AMP-protein kinase, which senses cellular energy status, is a key regulator in mediating many metabolic and stress response pathways, including fatty acid metabolism and oxidation, lipid metabolism, p53-mediated signaling and mammalian target of rapamycin (mTOR) signaling for protein synthesis [46], [47]. Of these pathways, the lipid/fatty acid metabolism–related pathways were significantly enriched in our pathway analysis. Detailed annotation of the upstream genes mediating AMPK activity and the downstream genes regulated by AMPK identified dramatic differential expression of genes in the AMPK pathway, in particular between the cells at other statuses compared with the M2-IL4 status (Fig. 5). Although antiviral regulation through modulation of AMPK-mediated pathways was recently reported [46], [47], the direct involvement of AMPK signaling in PRRSV infection has not been studied. Our further functional validation clearly indicated that anti-PRRSV activity can be elicited through drugs that modulate AMPK signaling (Fig. 5). Immunometabolism is a new front at the interface of immunology and metabolism that focuses on the integration and interaction of immune and metabolic systems in mediation of the development of diseases [57]. The regulation of AMPK-signaling and potential downstream lipid metabolism may posit new strategies for potentiating antiviral responses and for optimizing current vaccine strategies against PRRSV infection.

Epigenetic regulation, which involves chemical modification of DNA cytosine residues and DNA-bound histone proteins without alteration of DNA sequence, is emerging as one of the major factors regulating gene expression in response to environmental stimuli [44], [45]. Recent studies have demonstrated that epigenetic mechanisms have the potential to mediate activation status, including the antiviral state in monocytic cells. For example, dimethylation of histone H3 at lysine 9 (H3K9me2) has been shown to elicit suppression of IFN and IFN-inducible antiviral gene expression [56]. On the basis of family-wide annotation and DEG analysis of genes pertinent to epigenetic regulation, our RNA-Seq data identified more than a dozen epigenetic genes that were strikingly differentially expressed in PRRSV-infected macrophages at different activation statuses. Indeed, suppression of DNA and histone methylation, and particularly histone deacetylation, effectively inhibited PRRSV infection in cells.

In summary, in addition to identifying potential signature genes (Fig. 1, 2), our pathway analysis discovered multiple pathways (Fig. 4–6) significantly involved in response to PRRSV infection in macrophages at different activation statuses. It is noteworthy that our RNA-Seq analysis using polarized macrophages revealed a dozen more significant pathways that have not been reported in previous transcriptomic analyses using PRRSV-infected tissues or cells [27]–[34]. Furthermore, analysis of two key signaling pathways, AMPK-mediated and epigenetic mechanisms, not only clustered the pathway-inclusive DEGs pertaining to each activation status, but also functionally validated the involvement of AMPK-mediated and epigenetic pathways in regulation of antiviral response to PRRSV infection in cells. The major gene response pathways and functional determination of potential signature genes discovered here may lead to pathway-targeted design of better adjuvants for current vaccines and elicit the discovery of therapeutic measures using monocytic cells manipulated for antiviral propensity [17].

## Supporting Information

Figure S1 **(A) Quality control/assurance analyses of RNA-Seq reads.** Shown are diagrams of the statistics of raw reads, of which >99% are clean reads, and the distribution of genes’ coverage. Both diagrams represent analyses of the RNA-Seq data from the control sample (M0-PBS, see abbreviation below), which are comparable among all samples of MФs at different activation statuses (also see the supporting results of DEG statistics under the title of Table S2). **(B)** Statistics of differentially expressed genes [DEGs, FDR (false discovery rate) ≤0.001 and log2 Ratio ≥1] detected compared in each pair of samples. Abbreviations: M0-PBS, MФs mocked-treated with phosphate-buffered saline (PBS); M1-IFNγ, MФs at M1 status stimulated with IFN-γ; M1-LPS, MФs at M1 status stimulated with LPS; M2-IL4, MФs at M2 status stimulated with IL-4; M2-IL10, MФs at M2 status stimulated with IL-10; and MaV-IFNα, MФs at antiviral status stimulated with IFN-α.
(TIF)

Figure S2 **Verification of DEGs at the protein level using a proteomic procedure.** Equal amounts of proteins from macrophages at different activation statuses were stained with either red or green fluorescent dyes and co-resolved using a 2D-DIGE procedure (Applied Biomics, Inc., Hayward, CA) to isolate protein spots that significantly increased in macrophages at different activation statuses and to further identify isolated proteins by nano LC-MS/MS. The analytic 2D-DIGE gel is shown with overlapping protein samples from cells at both M1-IFNγ and M0-PBS statuses.
(TIF)

Figure S3 **(A-C) Illustration of DEGs in AMPK-mediated pathways in M1-IFNγ, M2-IL10, and MaV-IFNα activation statuses, respectively.** Color legends of the boxes framing gene symbols are shown as in Figure 5. (D & E) **S**uppression of PRRSV infection by two AMPK-pathway activators, salicylic acid (SA) and U18666, at physiological concentrations in MARC-145 cells and porcine monocyte-derived dendritic cells (mDCs). The fluorescent micrographs (inset) show cells infected by a GFP-labeled PRRSV, whereas the larger bright-field images show cell phenotypes with non-visible cytotoxic effects. The micrographs represent one of three replicates with similar results.
(TIF)

Table S1 **PCR primers used for RT-PCR assay of porcine IRF and IL-17 genes.**
(DOCX)

Table S2 **Collective results of sequencing assessment for quality control of the RNA-Seq data to meet the criteria for genome-wide transcriptomic analysis.**
(PDF)
