# Supplementary figures and images for: Genome-Wide Analysis of Antiviral Signature Genes in Porcine Macrophages at Different Activation Statuses
Source: PLoS One. 2014 Feb 5;9(2):e87613. doi: 10.1371/journal.pone.0087613 (PMC3914820; doi:10.1371/journal.pone.0087613)

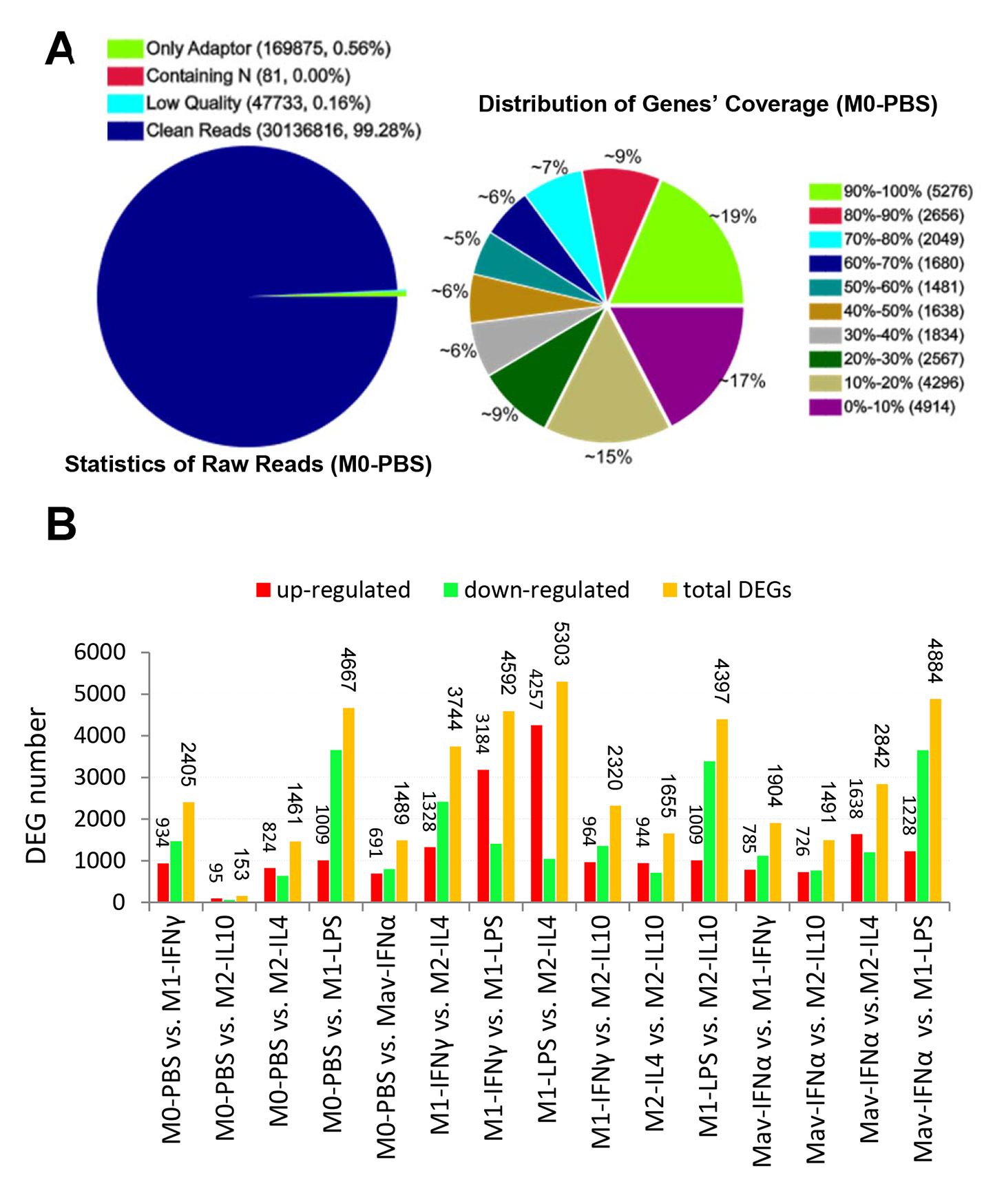

Supplement: Figure S1 — (A) Quality control/assurance analyses of RNA-Seq reads. Shown are diagrams of the statistics of raw reads, of which >99% are clean reads, and the distribution of genes’ coverage. Both diagrams represent analyses of the RNA-Seq data from the control sample (M0-PBS, see abbreviation below), which are comparable among all samples of MФs at different activation statuses (also see the supporting results of DEG statistics under the title of Table S2). (B) Statistics of differentially expressed genes [DEGs, FDR (false discovery rate) ≤0.001 and log2 Ratio ≥1] detected compared in each pair of samples. Abbreviations: M0-PBS, MФs mocked-treated with phosphate-buffered saline (PBS); M1-IFNγ, MФs at M1 status stimulated with IFN-γ; M1-LPS, MФs at M1 status stimulated with LPS; M2-IL4, MФs at M2 status stimulated with IL-4; M2-IL10, MФs at M2 status stimulated with IL-10; and MaV-IFNα, MФs at antiviral status stimulated with IFN-α. (TIF) [file pone.0087613.s001.tif]

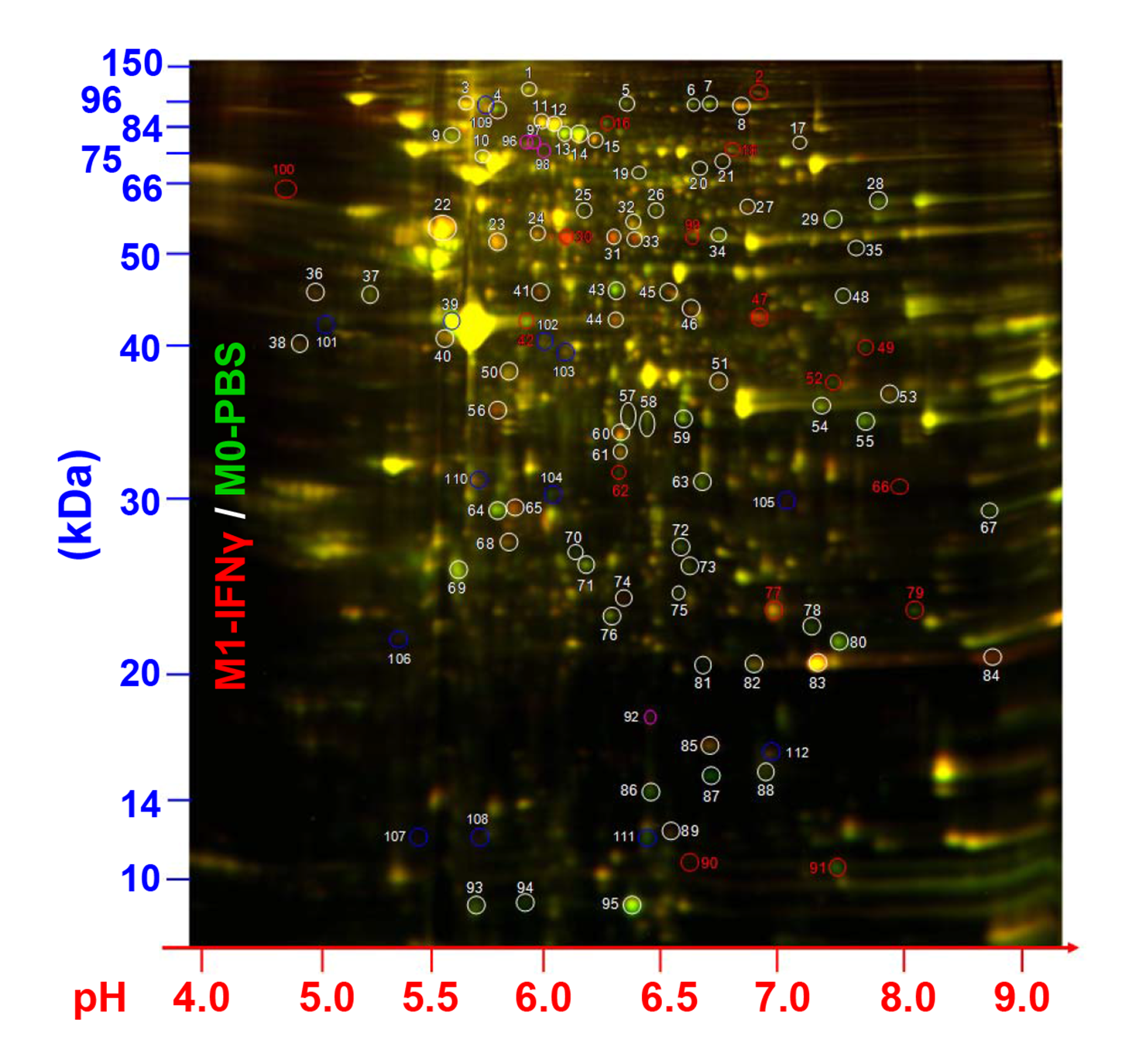

Supplement: Figure S2 — Verification of DEGs at the protein level using a proteomic procedure. Equal amounts of proteins from macrophages at different activation statuses were stained with either red or green fluorescent dyes and co-resolved using a 2D-DIGE procedure (Applied Biomics, Inc., Hayward, CA) to isolate protein spots that significantly increased in macrophages at different activation statuses and to further identify isolated proteins by nano LC-MS/MS. The analytic 2D-DIGE gel is shown with overlapping protein samples from cells at both M1-IFNγ and M0-PBS statuses. (TIF) [file pone.0087613.s002.tif]

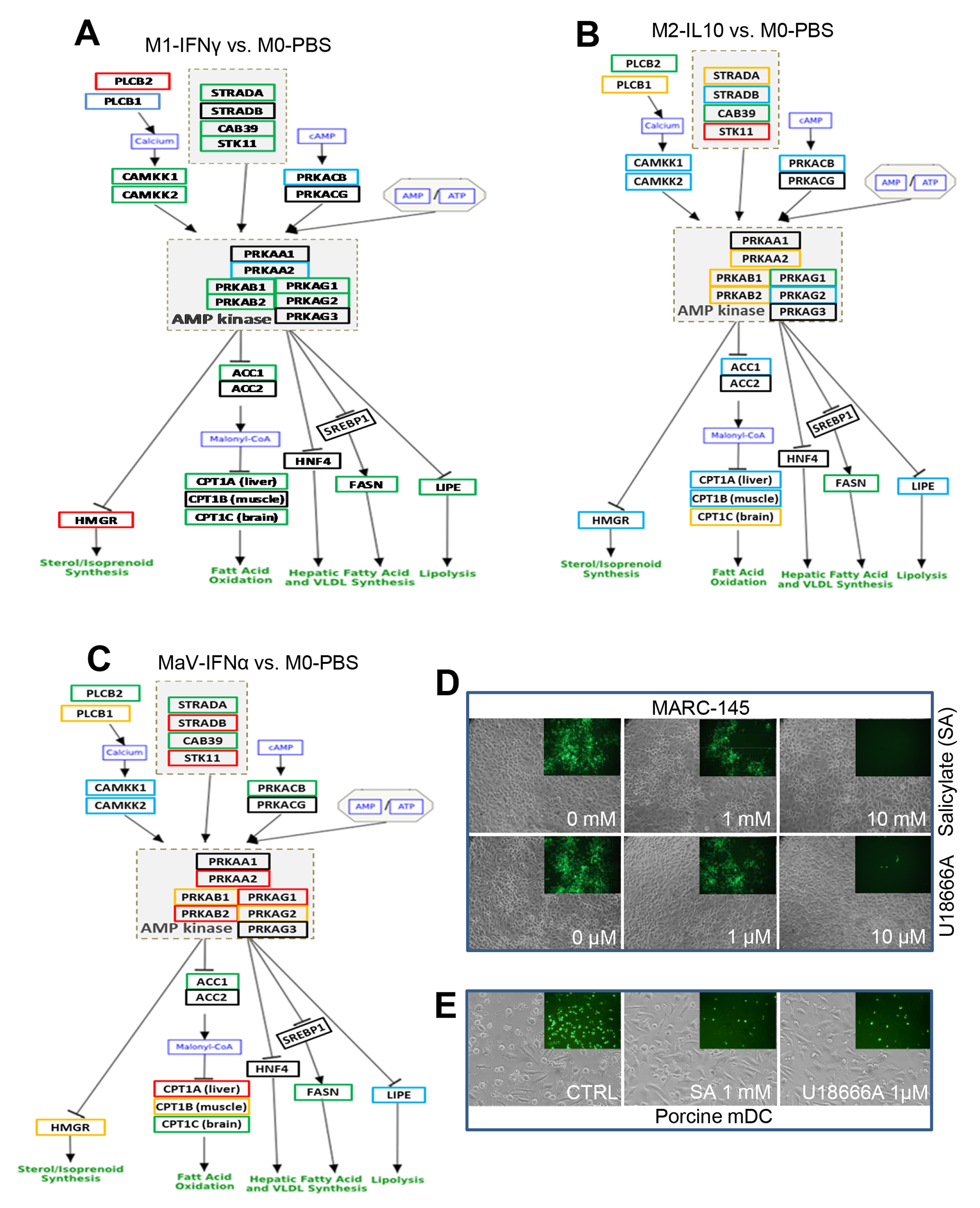

Supplement: Figure S3 — (A-C) Illustration of DEGs in AMPK-mediated pathways in M1-IFNγ, M2-IL10, and MaV-IFNα activation statuses, respectively. Color legends of the boxes framing gene symbols are shown as in Figure 5. (D & E) Suppression of PRRSV infection by two AMPK-pathway activators, salicylic acid (SA) and U18666, at physiological concentrations in MARC-145 cells and porcine monocyte-derived dendritic cells (mDCs). The fluorescent micrographs (inset) show cells infected by a GFP-labeled PRRSV, whereas the larger bright-field images show cell phenotypes with non-visible cytotoxic effects. The micrographs represent one of three replicates with similar results. (TIF) [file pone.0087613.s003.tif]
