# Supplementary material for: Genome-Wide Analysis of Antiviral Signature Genes in Porcine Macrophages at Different Activation Statuses
Source: PLoS One. 2014 Feb 5;9(2):e87613. doi: 10.1371/journal.pone.0087613 (PMC3914820; doi:10.1371/journal.pone.0087613)
Supplement: Table S1 — PCR primers used for RT-PCR assay of porcine IRF and IL-17 genes. (DOCX) [file pone.0087613.s004.docx]

**Table S1:** PCR primers used for RT-PCR assay of porcine IRF and IL-17 genes.

| IRFs | Primer sequence (5′-3′) | Product size (bp) | GenBank RefSeq RNA Accession |
| --- | --- | --- | --- |
| IRF1 | Forward: GCCCATCACTCGGATGCGCA  Reverse: TCCGGTACACCCGCACAGCT | 329 | NM_001097413.1 |
| IRF2 | Forward: GAGCGATGAGCAGCCGGTCA  Reverse: TCCGCGTCCCCATGTTGCTG | 185 | XM_003133334.1 |
| IRF3 | Forward: CGGTCTGCCCTGAACCGGAAA  Reverse: TGCATCCGAGGCCAAGTCCAT | 219 | NM_213770.1 |
| IRF4 | Forward: GGCTCCGCTGTGCCCTCAAC  Reverse: GGCACCATCGGACACGAGCC | 110 | XM_003359940.1 |
| IRF5 | Forward: TCCAGAGGATGTTACCAAGCCTGA  Reverse: ACTTGATCTCCAGGTCGGTCAAAG | 301 | XM_003134683.1 |
| IRF6 | Forward: AGGGCTCCGTCGTCAACCCA  Reverse: CCGCTTTGGGCCACACTGCT | 228 | NM_214278.1 |
| IRF7 | Forward: CCCACGCGTGCTCTTCGGAG  Reverse: GTGCAGGGCACAGCGGAAGT | 271 | NM_001097428.1 |
| IRF8 | Forward: GGGCGCTCGGAGATCGAGGA  Reverse: CTCGGGCCCGTACAGCTTGC | 321 | NM_001252427.1 |
| IRF9 | Forward: AAGGCCTGGGCGATATTTAAGGGA  Reverse: GTGGCGTCGCTTCGATGGTAATTT | 228 | NM_001078670.1 |
| IL-17A | Forward: ACCGGAGCACACCTGCCAGA  Reverse: GGGGGTGACACAGGTGCAGC | 284 | NM_001005729.1 |
| IL-17B | Forward: AAGCCGTTTGCCCGCATGGA Reverse: TGAACACGGGCACGCTCACC | 274 | XM_003124086.2 |
| IL-17D | Forward: TGCTCGTGCCTGCTGTCGTG  Reverse: TTGTCGGTTGCACCGTCCCG | 228 | EW441377 |
| IL-17E | Forward: TCCCCACTGGCCCAACTGCT  Reverse: CCTGGGGCAGCCGGTTCAAG | 212 | XM_003128538.1 |
| IL-17F | Forward: ACCGATCCAGCTCCCCCTGG  Reverse: CTTCCTGCCCCTGGGCGTTG | 117 | XM_001924366.2 |
| GAPDH | Forward: TGGYATCGTGGAAGGRCTCAT  Reverse: RTGGGWGTYGCTGTTGAAGTC | 370 | NM_001206359.1 |
