# Supplementary material for: Genome-Wide Analysis of Antiviral Signature Genes in Porcine Macrophages at Different Activation Statuses
Source: PLoS One. 2014 Feb 5;9(2):e87613. doi: 10.1371/journal.pone.0087613 (PMC3914820; doi:10.1371/journal.pone.0087613)

RNA-Seq data met the criteria for genome-wide transcriptomic analysis

During PRRSV infection in pigs, the virus exploits both alveolar macrophages and other tissue macrophages as primary infection sites for replication and spread. We have observed that porcine MΦs at different activation statuses differ dramatically in their permissiveness to PRRSV infection [17,36]. This observation, coupled with the pivotal roles of activated MΦs in regulating antimicrobial immunity [1-5], prompted us to conduct genome-wide analysis of signature genes pertinent to different activation statuses upon viral infection. Several analyses were conducted for quality control and to ensure that RNA-Seq data met the criteria for genome-wide transcriptomic analysis [48,49]. First, we conducted sequencing assessment for the quality and genome/gene mapping results of our RNA-Seq reads. As illustrated in Supplemental Fig 1A and Excel data sheets (Sheet 1 and 2, in next pages), the majority (>99.25%) of raw reads of each sample were clean reads in which ~80% clean reads could be mapped to current genome assembly or gene annotations. Unmapped reads (~20%) in each sample, which could have resulted from unknown transcripts or sequencing errors, were within the threshold of RNA-Seq procedures [49]. Sequencing saturation analysis demonstrated that a saturated stage of identified gene numbers was reached with more than 20 million (M) reads [49] and obtained with about 30 M reads of each sample (Sheet 3). Distribution statistics of reads mapped to reference genes were derived to demonstrate the relative randomness of reads along mapped reference genes, which was comparable among all samples (Sheet 4). We analyzed the distribution of reads mapped to the swine reference genome and drew the distribution of reads in the longest 25 chromosomal Scaffolds (Sheet 6). The genome distribution of reads along the analyzed scaffolds was correlated with the identified gene numbers and gene densities therein. In addition, reads mapped to more than 50% of transcripts flanked near 50% of their full-length sequences, as shown in supplemental data (Figure S1, and Sheet 5). Thus, our RNA-Seq reads met the criteria for genome-wide transcriptomic analysis in each sample and were comparable among all samples for analysis of DEGs and gene response pathways in PRRSV-infected MΦs at different activation statuses.

Sheet 1: Sequencing Quality Evaluation

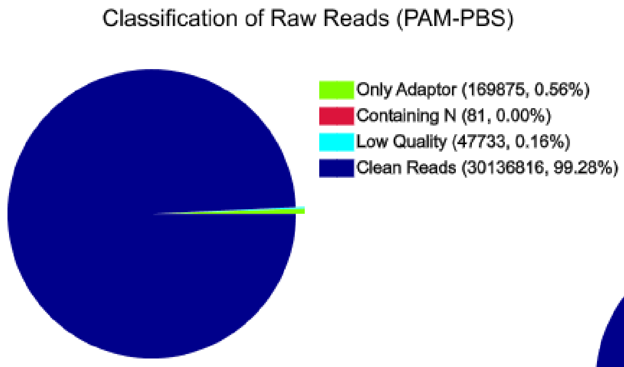

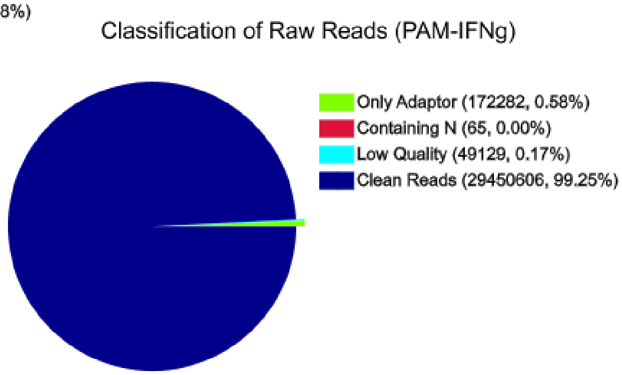

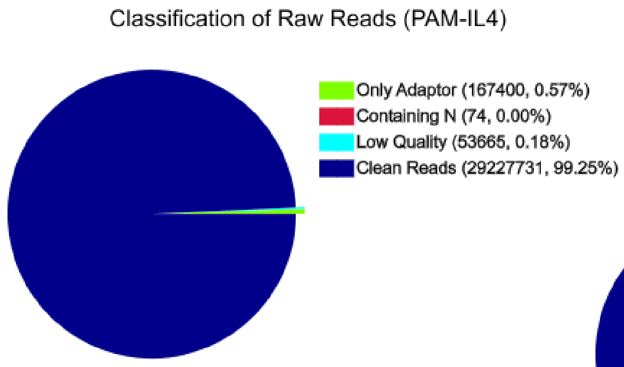

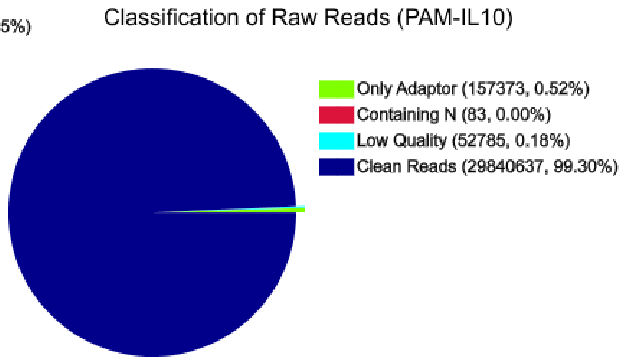

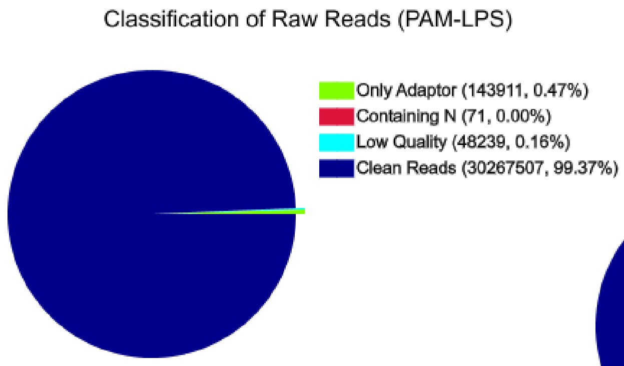

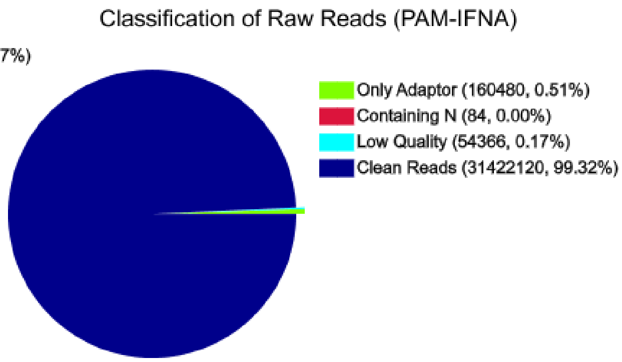

Sheet 2: Alignment statistics of the control sample, with a comparable statistics of other samples (not shown)

M0 (PAM-PBS): Stat. of Map to Genome

Map to Genome	reads number	percentage
Total Reads	30136816	100.00%
Total BasePairs	1476703984	100.00%
Total Mapped Reads	23965440	79.52%
perfect match	18641475	61.86%
<=3bp mismatch	5323965	17.67%
unique match	21003126	69.69%
multi-position match	2962314	9.83%
Total Unmapped Reads	6171376	20.48%

M0 (PAM-PBS): Stat. of Map to Gene

Map to Gene	reads number	percentage
Total Reads	30136816	100.00%
Total BasePairs	1476703984	100.00%
Total Mapped Reads	23997816	79.63%
perfect match	19310737	64.08%
<=2bp mismatch	4687079	15.55%
unique match	17734007	58.84%
multi-position match	6263809	20.78%
Total Unmapped Reads	6139000	20.37%

Sheet 3: Sequencing Saturation Analysis

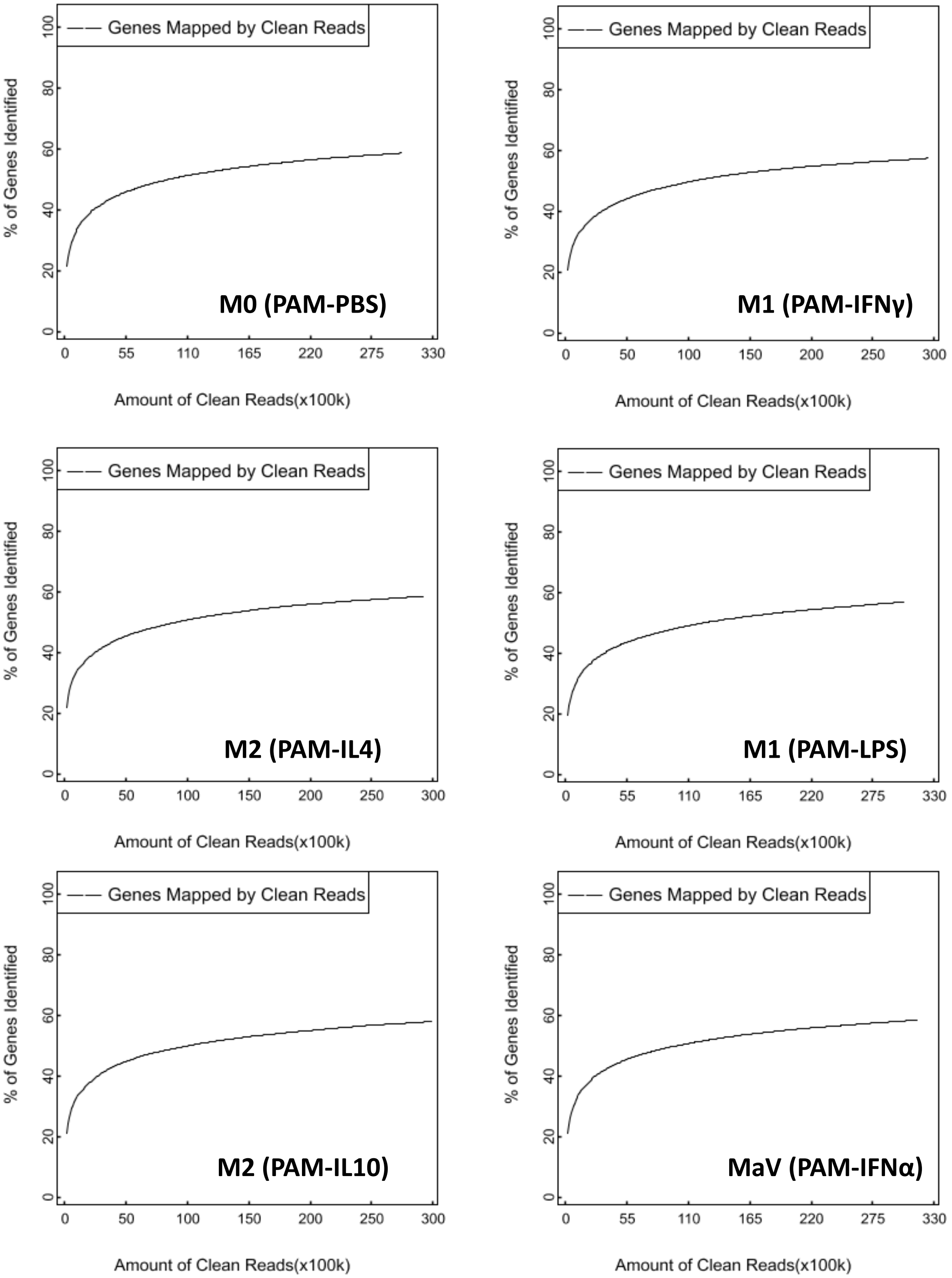

Sheet 4: Randomness assessment: Distribution statistics of reads mapped to reference gene

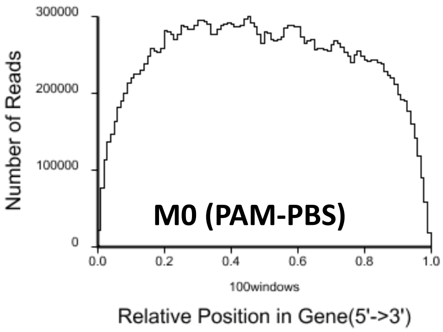

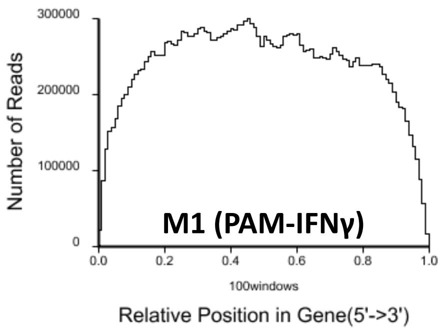

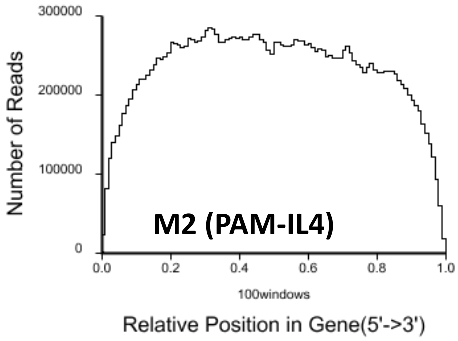

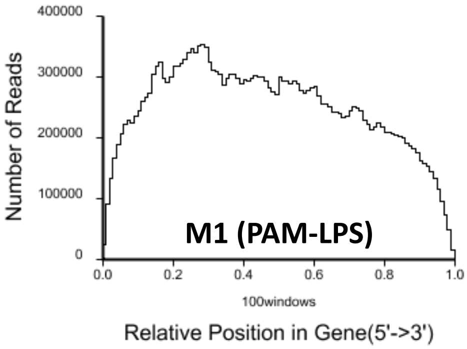

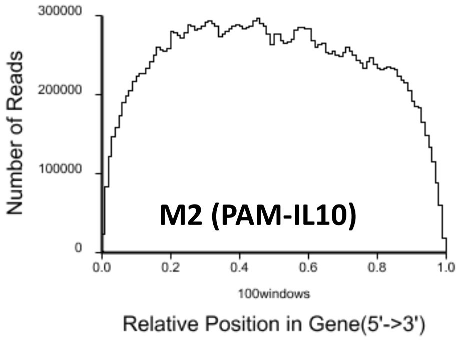

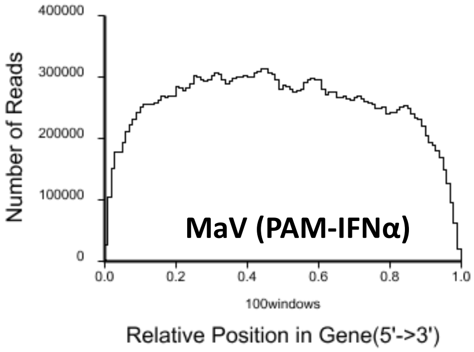

Sheet 5: Genome distribution: Shown is the consistence among statistics of gene numbers, coverage and RNA-Seq read numbers along three Chromosomal Scaffolds of 25 analyzed.

NC_010443.4	
Size (bp):	315321322
Chromosome:	1
Gene/exon whithin:	2395
Gene density/Kbp:	7.5954E-09

NC_010452.3	
Size (bp):	79102373
Chromosome:	10
Gene/exon whithin:	583
Gene density/Kbp:	7.3702E-09

NC_010461.4	
Size (bp):	144288218
Chromosome:	X
Gene/exon whithin:	978
Gene density/Kbp:	6.7781E-09

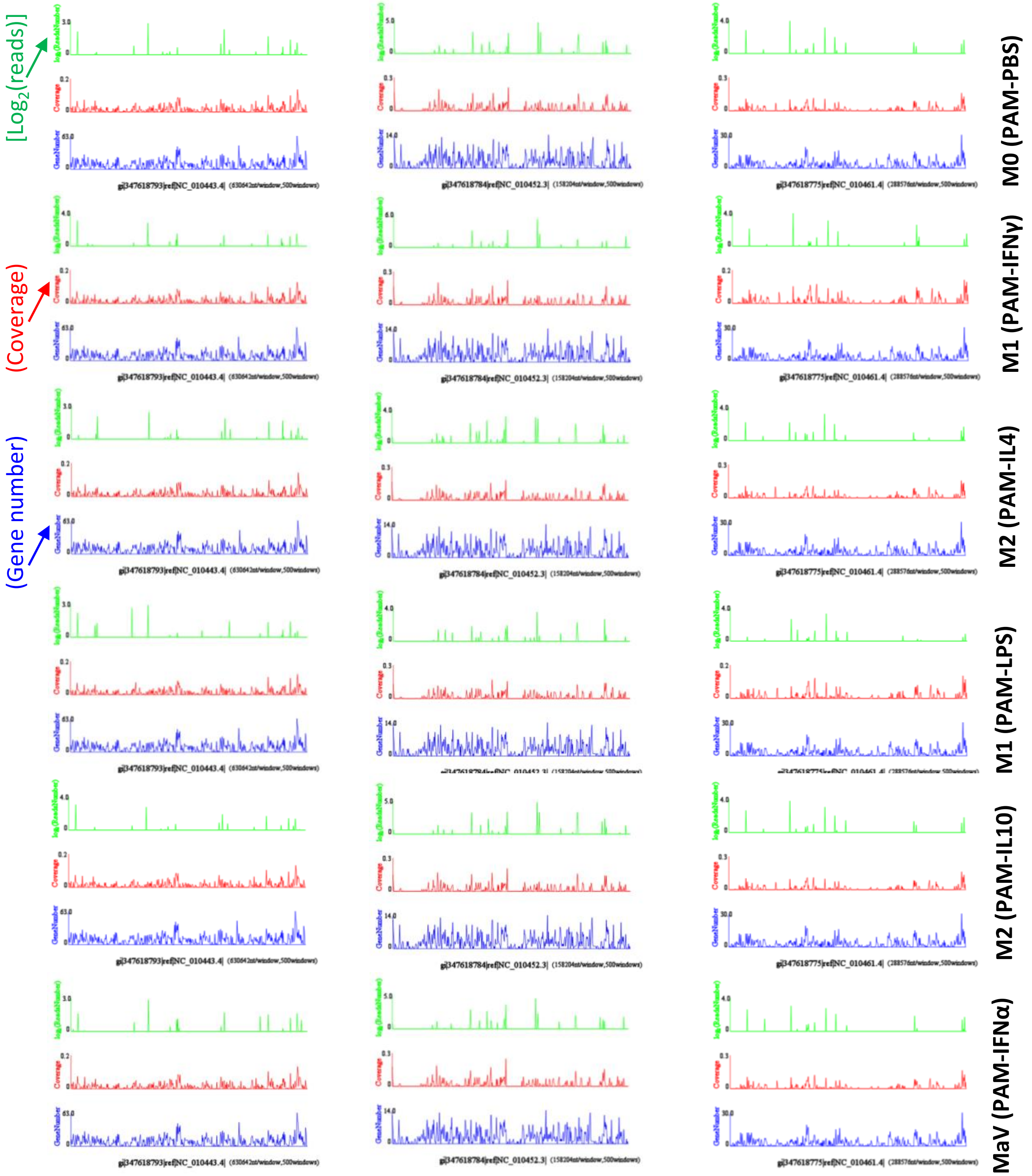

Sheet 6: Distribution of read coverage in transcripts detected

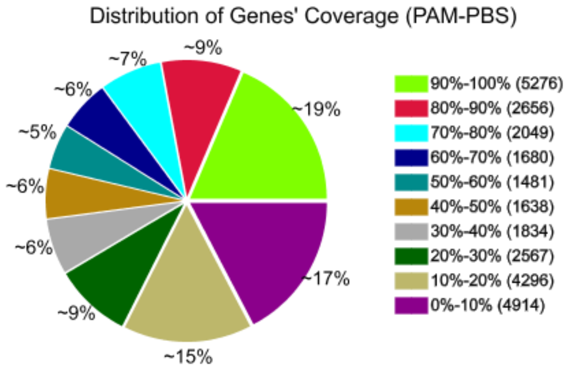

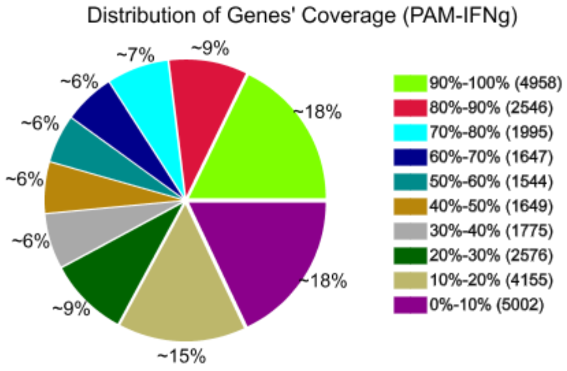

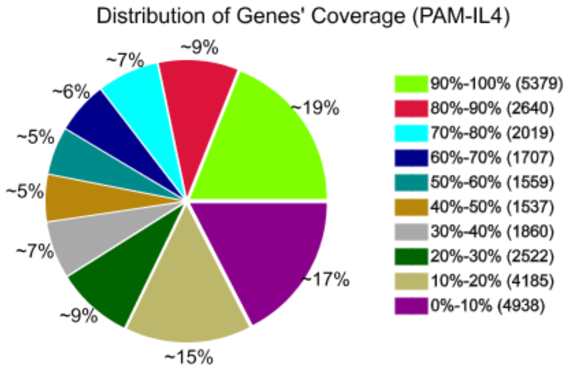

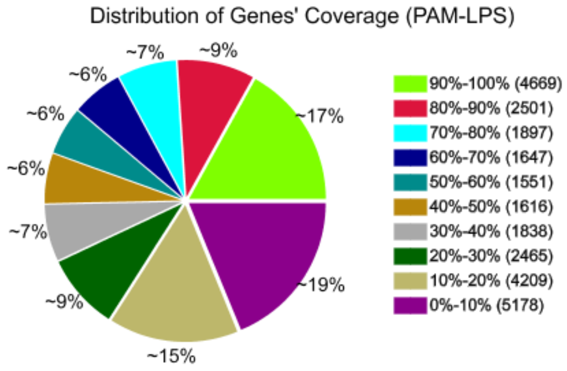

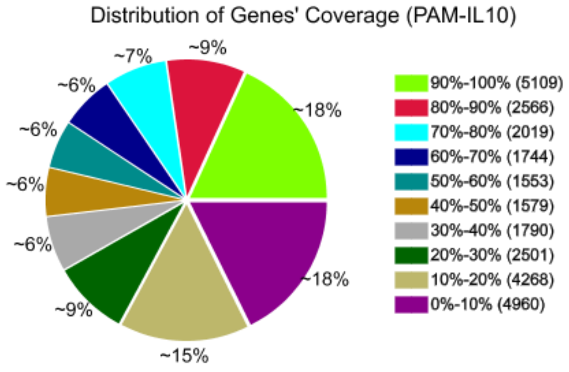

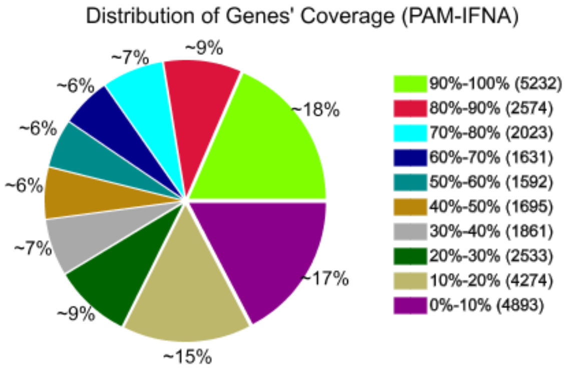

Supplement: Table S2 — Collective results of sequencing assessment for quality control of the RNA-Seq data to meet the criteria for genome-wide transcriptomic analysis. (PDF) [file pone.0087613.s005.pdf]
